# Tandem repeat copy-number variation in protein-coding regions of human genes

**DOI:** 10.1186/gb-2005-6-8-r69

**Published:** 2005-07-28

**Authors:** Colm T O'Dushlaine, Richard J Edwards, Stephen D Park, Denis C Shields

**Affiliations:** 1 Bioinformatics Core, Department of Clinical Pharmacology and Institute of Biopharmaceutical Sciences, Royal College of Surgeons in Ireland, 123 St Stephen's Green, Dublin 2, Ireland

## Abstract

**Background:**

Tandem repeat variation in protein-coding regions will alter protein length and may introduce frameshifts. Tandem repeat variants are associated with variation in pathogenicity in bacteria and with human disease. We characterized tandem repeat polymorphism in human proteins, using the UniGene database, and tested whether these were associated with host defense roles.

**Results:**

Protein-coding tandem repeat copy-number polymorphisms were detected in 249 tandem repeats found in 218 UniGene clusters; observed length differences ranged from 2 to 144 nucleotides, with unit copy lengths ranging from 2 to 57. This corresponded to 1.59% (218/13,749) of proteins investigated carrying detectable polymorphisms in the copy-number of protein-coding tandem repeats. We found no evidence that tandem repeat copy-number polymorphism was significantly elevated in defense-response proteins (*p *= 0.882). An association with the Gene Ontology term 'protein-binding' remained significant after covariate adjustment and correction for multiple testing. Combining this analysis with previous experimental evaluations of tandem repeat polymorphism, we estimate the approximate mean frequency of tandem repeat polymorphisms in human proteins to be 6%. Because 13.9% of the polymorphisms were not a multiple of three nucleotides, up to 1% of proteins may contain frameshifting tandem repeat polymorphisms.

**Conclusion:**

Around 1 in 20 human proteins are likely to contain tandem repeat copy-number polymorphisms within coding regions. Such polymorphisms are not more frequent among defense-response proteins; their prevalence among protein-binding proteins may reflect lower selective constraints on their structural modification. The impact of frameshifting and longer copy-number variants on protein function and disease merits further investigation.

## Abstract 2

Tandem repeat polymorphisms in human proteins were characterized using the UniGene dataset. This analysis suggests that 1 in 20 proteins are likely to contain tandem repeat copy-number polymorphisms within coding regions; these were prevalent among protein-binding proteins.

## Background

DNA tandem repeats are two or more adjacent and approximate copies of a sequence of nucleotides. The presence of tandem repeats has been associated with a number of diseases and phenotypic conditions. For instance, repeat polymorphisms in 5' and 3' regions are known to cause diseases such as Huntington's disease [1] and certain forms of Fragile X syndrome [2]. Other tandem repeat polymorphisms in noncoding regions are known to modify function through their impact on gene regulation [3,4]. These polymorphisms can arise from events such as unequal crossover, replication slippage or double-strand break repair [5-7].

Polymorphism of tandem repeats within protein-coding sequences is known to modulate disease risks and can effect changes in the protein products of genes, leading to diseases such as myotonic dystrophy [8]. A number of diseases caused by repeat polymorphism arise from the expansion of trinucleotide repeats [9]. Other longer repeat polymorphisms have been postulated to modify disease risk (for example, platelet glycoprotein Ib-α (*GP1BA*) repeat [10], the kringle repeat in apolipoprotein(a) (*LPA*) [11], and P-selectin ligand (*SELPLG*) repeat [12]).

While single-nucleotide polymorphisms (SNPs) are currently the subject of extensive research, tandem repeats can exhibit high levels of length polymorphism that will potentially alter protein function. In addition, the comparatively greater mutability of certain classes of tandem repeats may lead to a different spectrum of effects on function, as mildly deleterious variants of recent origin may not have had time to be eliminated. Previous studies [13,14] have predicted polymorphism using a minimum threshold of repeating units and a minimum homogeneity criteria. The threshold refers to the minimum number of repeat units needed for a locus to be scored as likely to contain polymorphism, and the homogeneity refers to percentage of nucleotides within a repeat that may deviate from the core repetitive unit. The criteria depended on the length of the repeat unit and were drawn from the literature on repeat polymorphisms. For instance, for a dinucleotide repeat block to be scored as a likely polymorphism, a threshold number of eight repeat units and a minimum homogeneity of 0.9 was required.

This approach was used to predict 11,265 potentially polymorphic tandem repeats and led to the proposal that 22% of UniGene [15] clusters contain at least one potentially polymorphic locus [14]. Of these, 8% were predicted to be in coding regions. If polymorphic, these loci could cause frameshift mutations, which would be likely to significantly alter the protein product. However, these studies only analyzed a single representative sequence from each UniGene cluster, and did not investigate the observed variability among all sequences within the cluster. Additional studies predicting potentially polymorphic repeats have focused on minisatellite repeats. For instance, Denoeud and colleagues [16] were more interested in highly polymorphic minisatellites and only used strict definitions of minisatellites (unit length greater than 17 nucleotides, for instance). Naslund and co-workers [17] used a logistic regression approach to predict potentially polymorphic repeats. However, they were specifically interested in minisatellites with a minimum repeat unit length of six nucleotides and not the full spectrum of repeat unit lengths. Denoeud and Vergnaud have carried out genomic comparisons of related bacteria to observe tandem repeat sequence length differences [18]. However, no such analysis has been carried out to detect human repeat polymorphism.

It has been hypothesized that an excess diversity of coding tandem repeats contributes to antigenic variation within the prokaryotic pathogen *Neisseria *[19]. Variations in the numbers of repeats within the collagen-like region in *Bacillus anthracis *correlated with variation of filament length on the spore surface and have been proposed to affect the properties of the spores in response to various environments [20]. Indeed, repeat-mediated variation may form an integral part of the ability of many pathogens to adapt and remain adapted to their hosts and environments [21] and has been proposed as a molecular basis for the rapid adaptation of both prokaryotes and eukaryotes to environmental changes [22]. Our investigations sought to find evidence of the existence of this in humans. We proposed that repeat polymorphism within host-defense proteins in the human population might be advantageous, as previously postulated [14], and thus we would expect higher levels of tandem repeat sequence length variation in these genes. Such proteins exhibit rapid rates of evolution in interspecies comparisons, consistent with positive selection for changes in response to pathogen selection pressures [23,24].

Here we report an investigation into the level of apparent polymorphism in human genes within the UniGene database, and examine whether such polymorphism is elevated in host-defense genes.

## Results and discussion

### Protein-coding repeat distribution

Of the 106,937 UniGene [15] sequence clusters, 14,953 (14%) contained coding sequence annotation. Of these, a total of 13,749 (13%) clusters had more than one sequence overlapping a repeat block, enabling a search for tandem repeat copy-number variants.

**Figure 1:**
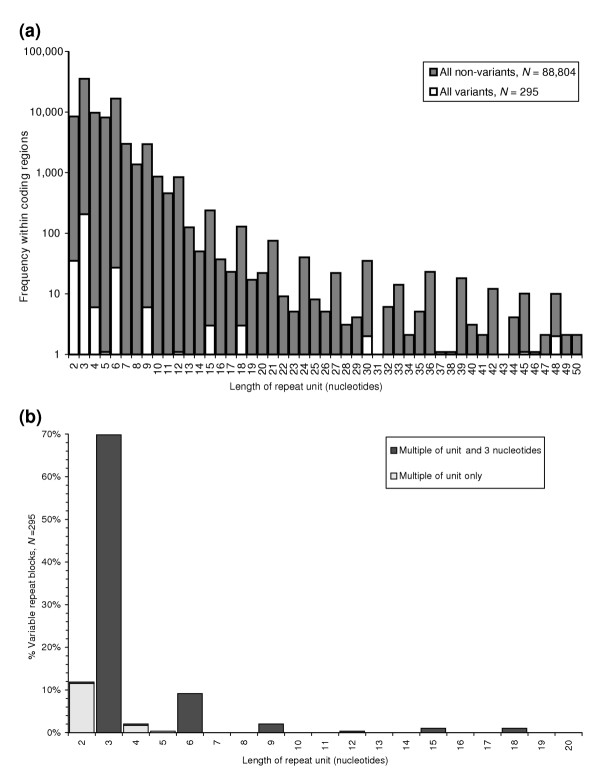
Frequency of variant and invariant repeats. **(a) **Histogram of the frequencies of different length repeat units in the dataset. Repeats that are multiples of three occur at greater frequency across both variant and non-variant repeats. Mononucleotide repeats were not included in the analysis. Variants represent differences between the representative and the alleles that are a multiple of the unit length and consistent with a change in repeat copy-number. *N*, number of identified length variants (295 variants observed in 249 tandem repeats in 218 genes). For the non-variant repeats, *N *represents the number of unique invariant repeats. The *x*-axis is on a logarithmic scale. **(b) **Breakdown of repeat variants by the type of variant. Unit lengths 2 to 20 are shown here, encompassing 288 of the 295 variants. Areas in black above bars 2 and 4 represent variants of units this length that are also a multiple of three.

A total of 89,243 tandem repeats were detected in protein-coding regions of the 13,783 UniGene representative sequences. The detected repeats were predominantly those with short repeat unit lengths of two to six nucleotides (Figure 1a). The distribution showed a clear elevation of repeat units that were a multiple of three, which agrees with previous findings that protein-coding region repeats whose copy-number variation is likely to cause frameshift errors occur at a lower frequency in coding regions [25-27]. We noted a much greater excess of trimer repeats relative to dimers and tetramers in this dataset than in a previous genomic analysis of exonic sequences [27]. This is likely to largely reflect the exclusion of 5' and 3' untranslated regions (UTRs) from our dataset; intronic and intergenic regions in the genomic analysis had a still greater incidence of dimers and tetramers compared to trimers [27]. Thus, although there is an apparent mutational bias against trimer repeats in genomic sequences, within protein-coding regions they are the most frequent class of tandem repeats. Of the detected repeats 82% were 100% homogenous. Thus, 18% of the dataset included were inexact repeats, with a higher proportion of inexact repeats among the arrays composed of longer repeat units.

### Range of tandem repeat copy-number variation

Detected variants were screened to ensure that they represented length variation arising as copy-number differences in genomic DNA, rather than intron retention or alternative splicing. Only length variations that corresponded to a length difference that was a multiple of the repeat unit were selected. This reduced the number of clusters with variation from 4,458 (16,483 query/hit pairs) to 623 (3,111 query/hit pairs). For this set, tandem repeats were detected in the variant sequence and checked to ensure that the observed copy-number was in agreement with the expected one, given the length of the hit block and the length of the repeat unit, further reducing the dataset to 218 clusters with observations of length variation (753 query/hit pairs).

In total, 249 unique repeat blocks (spanning 218 clusters) showed variation that was consistent with a change in repeat copy-number (Figure 1). We found 295 allelic variants that differed from the UniGene representative sequence (Additional data file 1) and 85.8% of these variants were a multiple of three nucleotides (253/295). Nearly 70% of variations that were a multiple of both three nucleotides and the repeat unit length arose within blocks of trinucleotide tandem repeats (Figure 1). Although some of the invariant repeats were imperfect, all the variant repeats were 100% homogenous (that is, every repeat unit was identical), and a large proportion were short (48% of variant repeat arrays were less than 20 nucleotides in length). The mean percentage match for repeats with array length less than 20 nucleotides was 98.52%. The mean percentage match for repeats with array length equal to or greater than 20 nucleotides was 90.50%.

**Figure 2:**
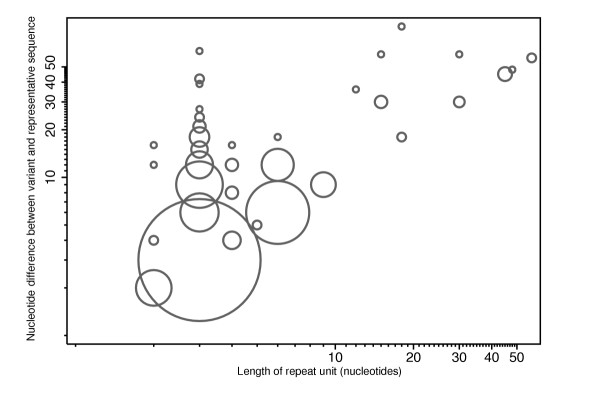
Weighted scatter-plot of the pattern of detected tandem repeat length variation. Length of repeat unit is plotted against the absolute difference between query and hit repeat block lengths. One variant corresponding to a length difference of 144 for a 48-nucleotide repeat has been omitted. Note that the length of repeat unit, rather than the tandem repeat array length, is plotted on the *x*-axis and most observed length differences are multiples of the corresponding unit length. The area of each circle is proportional to number of variants observed with a given unit length, and a given nucleotide difference between the representative and variant sequences.

Figure 2 illustrates the length differences observed between representative and other sequences. The majority of longer base differences were observed in repeats with a long repeat unit. Also, in most cases the majority of differences for a repeat of a given length are equal to one copy of that repeat, as indicated by the size of the circles in Figure 2. Among the longer repeat units, the variant alleles typically only differ by a single repeat unit (points along the diagonal). Allelic variants that differ by a larger number of repeat units are seen more often among the shorter repeats. The longest repeat units that exhibited polymorphism were 18 (3 representatives), 30 (2), 45 (1), 48 (2) and 57 (2) nucleotides in length (Figure 2, see also Additional data file 1). Of these large variants, the effects of the dopamine D4 receptor (*DRD4*) and *SELPLG *polymorphism have been well investigated [28,29], indicating probable effects on function and disease. The functional or clinical impact of the other variants remain to be evaluated, however. Clearly, the UniGene sampling approach is incomplete, and there are likely to be more large variant repeats in the human proteins; for example, the well known *GP1BA *polymorphism, with a unit size of 39 nucleotides, and the mucin 2 (*MUC2*) polymorphism [10,30]. These variants were not identified by this study, since the UniGene cluster sizes for these genes were too low to detect the common variants [31]. Three of the trimer repeats exhibited substantial length differences (39, 42 and 63 nucleotides, Figure 2), which are again likely to affect protein function. These were in the genes for the alpha 1A subunit of the voltage-dependent, P/Q type calcium channel (*CACNA1A*), the TATA-box binding protein (*TBP*) and the translocated promoter region to the activated *MET *oncogene (*TPR*) (Additional data file 1). While most of the *CACNA1A *allelic variants were in the 'normal' range of variation, the longest allele of 24 repeats was in the size range associated with the well studied trinucleotide-repeat expansion disease spinocerebellar ataxia 6 (*SCA6*) [32]. For *TBP *all eight allelic variants were below the length associated with a form of inherited ataxia [33,34]. *TPR *has not been associated with trinucleotide-repeat expansion diseases. A region of this oncogene has, however, been associated with nonrandom chromosomal deletions [35], and the role of this polymorphism in cancer may be of interest.

As an independent check for the completeness of our observations, the Human Gene Mutation Database (HGMD) [36] was queried with a set of all official HUGO gene symbols. A total of 18 contained coding-sequence repeat polymorphisms. Of these, eight (or 44%) were detected in our analysis - *HD*, *ATXN1*, *ATXN2*, *AR*, *CACNA1A*, *TBP*, *SELPLG*, and *ATN1*. Four of the remaining ten lacked coding-sequence annotation in the Hs.seq.uniq representative precluding the use of our method. One of the remaining six was a polymorphic mononucleotide repeat - these repeats were not included in our analysis. Two further genes contained cryptic GCN repeats. The last three had no variant hits in UniGene, either because of small cluster size (13, 170, 56), sequence error reducing the amount of hits (within-cluster alignments), or a lack of sufficient sequence coverage over the repeat region. Thus, in total, seven repeat variations were 'missed' either because of a lack of UniGene coding sequence annotation (4) or as a result of cluster size/sequence quality limitations (3), and three did not conform to the types of repeats considered in our analysis. Therefore, in relation to repeat variations previously associated with disease and considered in our analysis, we detected variations in 53% (8/15) of the associated genes.

This analysis highlights that fact that, while UniGene is a useful resource for looking at polymorphism, it has its limitations, specifically in relation to sample size, sequence quality and annotation. Of the 218 gene clusters with repeat variation, 34 had entries on the HGMD, eight of which - *HD*, *ATXN1*, *ATXN2*, *AR*, *CACNA1A*, *TBP*, *SELPLG*, and *ATN1 *- had coding-region repeat polymorphisms that were detected in our analysis. One further gene - *VWF *- was annotated as having a small deletion that corresponded to one of our repeat variants. Another gene - *TWIST1 *- was annotated as having a small deletion in the Saethre-Chotzen syndrome phenotype, which was detected in our analysis as a 12-nucleotide indel for a three-nucleotide repeat (GGC). While the variation observed in *VWF *may have arisen from a repeat slippage event, the variant for *TWIST1 *is unlikely to have done so. In addition to these variants, three genes - *NUMBL*, *E2F4 *and *NOTCH4 *- were annotated by Online Mendelian Inheritance in Man (OMIM) [37] as exhibiting trinucleotide repeat variation. Thus, 13 variants detected in our analysis were previously identified.

### Frequency of repeat variants

Given the likely sampling errors and biases, we did not expect frequencies of repeat variants to closely reflect true population frequencies. However, for known repeat variations from the literature that were also detected in our analysis, we compared heterozygosities by querying the GDB database [38]. For a set of five genes that had heterozygosity information and existed in the GDB database (*HD*, *AR*, *TBP*, *ATN1*, *HRC*), the heterozygosity in GDB was broadly similar (values of 0.8, 0.63, 0.81, 0.79 and 0.55, respectively) to that estimated from this dataset (Additional data file 2).

### Repeat copy-number and extent of variation

**Figure 3:**
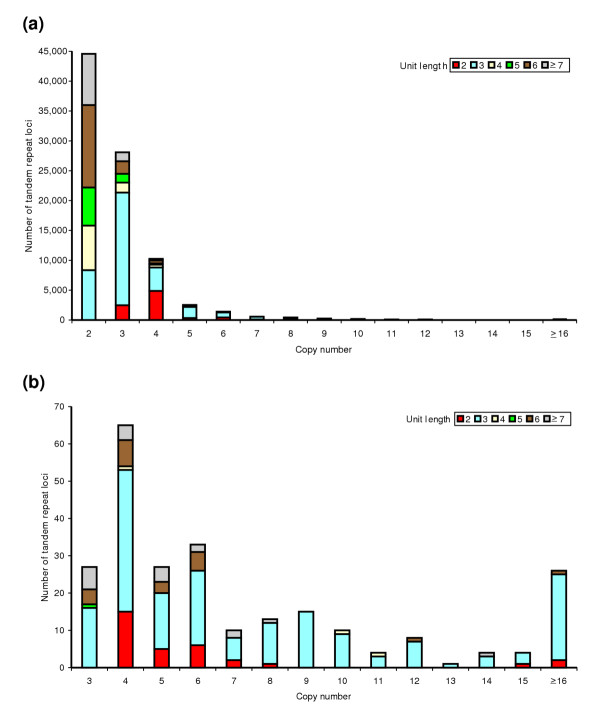
Distribution of copy-numbers of tandem repeats. The *x*-axis indicates the number of tandem repeat loci of a given unit length (indicated by color key) and with a given copy-number (indicated on the *x*-axis, rounded to the nearest whole number). **(a) **Non-variants, *N *= 88,850; **(b) **variants, *N *= 249; copy-number for variants represents the average copy-number among variants.

We compared the mean copy-number of the tandem repeats between clusters that have repeat variants and those without and found a significant difference (Mann-Whitney, *p *< 0.0001). As expected, the trend is for variant repeats to have a higher copy-number (Figure 3). This observation [39] has formed the basis of previous studies predicting repeat variation [13,14]. This difference in copy-number for the trimer repeats did not simply reflect a shift in the mean copy-number; there was a substantial upper tail in the distribution, indicating that the chance of a trimer being polymorphic increases as the copy-number increases. In contrast, there was no such marked tail of variants of relatively high copy-number for dimer repeats (Figure 3). This difference between dimer and trimer variation could represent a difference in mutational mechanisms, or, alternatively, the dimers may be subject to purifying selection against expansion, as most of the dimer variants are likely to cause frameshifts.

### Origin of variation

Interestingly, the vast majority of dimer, tetramer and pentamer copy-number variants resulted in a length difference that was not divisible by three (Figure 1b). Given the preference for repeat variation that is a multiple of three nucleotides, we had anticipated that there would be a greater proportion of copy-number variants that expand or contract dimer and tetramer repeats by exactly three copies (for example, we expected to see a larger number of dimer tandem variants that differed in length by six nucleotides). The observation that such variants are very rare (Figure 1b), even though they do not disrupt the reading frame, strongly supports the stepwise mutation model for microsatellite repeats [40,41], and suggests that insertion/deletion mutations of more than one unit at a time are quite unusual. It is probable that the frameshifting copy-number variants are mainly recent mutations that are selectively deleterious, reducing the chance of gradual expansion of the tandem array variant over time; trimer repeat variants could typically be much older. Thus, the majority of copy-number mutations in tandem arrays with short unit sizes are likely to arise by slippage [42], which occurs most often in homogenous repeats [43,44]. Consistent with this, the majority of observed variants for these repeats differ by a single unit.

In contrast, for a number of the larger tandem repeats (unit size of 12 and above) the observed variants in some cases differ by more than one copy, with no sampling of an intermediate allele (Figure 2). Such longer repeat variants may potentially arise through recombination, rather than slippage mechanisms, giving the potential for the gain or loss of more than one unit at a time. It should also be mentioned that the use of UniGene to detect variation precludes the ability to determine if the variation exists at DNA or transcriptional level. Our requirement that observed length variations had to be consistent with a change in repeat copy-number minimized the likelihood of detecting variation resulting from an alternative splice site arising within a repeat block. This did not, however, rule out inclusion of alternative splices where the splice sites might coincide with boundaries of tandem repeat units. Inspection of the intron/exon structure of genes in our results using EnsEMBL [45] revealed no such examples (data not shown).

### Frameshifting copy-number variation

This dataset is likely to underestimate the frequency of frameshifting repeat variants, as a large number of frameshifts stimulate nonsense-mediated RNA decay, biasing against their chance of being detected in UniGene. Messages carrying stop codons more than 50 nucleotides upstream of an intron are typically subject to rapid mRNA decay [46]. Secondly, nonsense polymorphisms typically occur at a low frequency in human proteins [47], reflecting selection against deleterious alleles, and it is possible that frameshifting tandem copy-number variants may similarly be at a lower frequency. Given the small sample size for many of the UniGene clusters, the incidence of frameshifting polymorphisms is probably strongly under-represented.

A few of the observed variants may not be true frameshifts, however, owing either to errors in coding-sequence annotation, sequencing errors, transcriptional errors or transcribed pseudogenes in the database. While we cannot definitively rule these out, the validation of repeat variants to ensure that they represent a change in repeat copy-number would reduce that possibility of some of these errors arising. Nevertheless, for the two reasons outlined above, we believe that the observation of one frameshifting tandem repeat polymorphism per 404 (34 out of 13,749) proteins surveyed (0.25%) represents a likely lower bound of the frequency. Wren *et al*. [14] predicted that 0.5% of proteins are likely to contain frameshifting tandem repeat polymorphisms.

It is of course possible that frameshifting tandem repeats can arise from sequencing errors, transcription errors or pseudogene transcripts. We inspected the 34 sequences containing frameshifting dinucleotide variants, and found that, in all but one sequence, the percentage of bases that were ambiguous (denoted by base 'N') was less than 1% (the outlier was 4%). We also searched the 51 frameshifting sequences and the representative allele against the human genome, and in each case both alleles hit the same sequence; that is, there was no evidence for the existence of a pseudogene with greater similarity to the frameshifted allele.

We cannot rule out the possibility of occasional transcriptional slippage giving rise to a small proportion of the observed variation: an experimental screen for such transcriptional errors estimated their frequency at approximately 1 in 5,000 transcripts in dinucleotide tandem repeats [48]: in our survey of 5,304 sequences containing 8,449 dinucleotide repeats, we found an incidence of 36 frameshifting dinucleotide mutations, compared with an expectation of less than two, arising from transcriptional errors. Secondly, two of the tetramer frameshifting repeats, and four of the dimer repeats, were observed in more than one sequence, which is a strong indication of a DNA, rather than a transcriptional, difference. None of the variants detected involved complete deletion of the repeat, with the lowest copy-number in the variant being 1.8 (see Additional data file 1).

### Association of copy-number variation and host-defense functions

While previous work has shown clear ontological trends for repeats that exhibit variation, it was restricted to certain classes of repeats [49]. We tested whether there was an excess of tandem repeat polymorphic variation in host-defense proteins by comparing the frequency of polymorphic genes among those classified as being related to 'defense response' (GO:0006952) [50] or not. There were 484 UniGene clusters that mapped to defense-response proteins and 8,129 clusters that did not. The mean variation was marginally higher in the defense-response category but this was not significant (*p *= 0.982, Chi-squared test) (Table 1).

The ability to detect repeat variation within a given cluster is partially dependent on both the number of sequences in which we detected tandem repeats, and the number of repeat blocks in the sequence. These are highly correlated with the number of sequences in the cluster and sequence length, respectively (data not shown). It is possible that these two variables - cluster size and sequence length - might relate to protein groupings with certain functions. In addition, cluster size may be affected by ascertainment bias for certain genes highly expressed in well sampled tissues, and there may be an ascertainment bias towards variant sequences that have been preferentially selected for sequencing. Therefore, we performed a logistic regression where the dependent categorical variable described whether or not the cluster contained a variant repeat population, and tested this against the categorical 'defense response' variable (describing whether the cluster linked to the GO term). We considered as covariates the number of sequences within each cluster as well as the length of the protein. We found that variation was not dependent on the defense-response classification when both the number of sequences and the length of the protein were considered as covariates (*p *= 0.882) (Table 1).

Thus, we find no evidence that human host-defense proteins have an excess of tandem repeat variation. It is possible that the large size of human gene promoters and their innate variability (in SNPs, tandem repeats, indels and other polymorphisms) provides ample opportunity in response to pathogen challenges for rapid selection of variants modulating gene function. There may therefore be no strong long-term selection pressure to develop an innate reservoir of potential variation within the protein sequences themselves. We anticipate that it may be more likely that such advantageous tandem repeat polymorphisms would arise in host-defense proteins of organisms that lack the adaptive immune system and have much larger population sizes.

### Association of tandem repeat copy-number variation and Gene Ontology (GO) terms

We investigated whether the occurrence of copy-number polymorphisms was associated with any other GO terms. Of the 362 level-4 terms in GO [50], 167 terms could be linked to our dataset and had at least one cluster linking to the term. We tested whether or not variation was significantly associated with any of these terms using a Fisher's exact test. This found 13 terms to be significant, of which only the term 'protein-binding' (GO:0005515) remained significant after Bonferroni correction for multiple testing. Again, we wished to ensure that the UniGene cluster size and the sequence length were not confounding the associations between variability and GO terms. Therefore, we performed the logistic regression described above, for which 67 of the 167 terms had a sufficiently large sample size to be tested. Twelve of these terms were significant, one of which remained significant after correcting for multiple testing. Again, this term was 'protein binding'. To ensure that the observed significance could not be largely attributed to differences in repeat copy-number between variants and non-variants (Figure 3) we performed the logistic regression with the mean repeat copy-number per cluster as an additional covariate. The significance remained the same under this model (*p *< 0.00001).

Length changes in repeats involved in protein-protein interactions may affect the evolution of cellular signaling pathways [51]. This process may be facilitated by an absence of selective constraint on the repeat if there are no deleterious effects on the phenotype. An elevation of sequence variability at the population level in these proteins is similarly consistent with lack of evolutionary constraint on the protein regions. Previous work has shown that for polyglutamine repeats between human and mouse, there is an association between new repeats and a high nonsynonymous sequence divergence rate, corresponding to regions of low purifying selection [52]. Further investigation of the classes of repeats that are polymorphic in different groups of genes is of interest [53] but sample sizes are too limited to draw strong inferences.

We investigated in more detail the 45 variant clusters linked to 'protein-binding'. Investigation of the daughter GO terms did not reveal any striking association with any subcategory (data not shown). A number of clusters corresponding to this category have previously been described to be associated with disease, particularly trinucleotide-repeat expansion diseases [54,55]. The existence of repeats in protein- and DNA-binding proteins has been linked to their functional roles [56-60]. The question is whether the polymorphisms in these repeats are likely to have a functional impact. There are two models that may explain the higher level of polymorphism. One is that these proteins are typically under low selective constraint, as repetitive regions in protein- and DNA-binding proteins are often substantially structurally disordered [60] and expansion is unlikely to destabilize the protein's overall folding. Supporting this is the observation that new repeats emerge in regions of proteins that are subject to lower-than-average levels of purifying selection [52]. The second model is that such polymorphisms are promoted by balancing selection or recent selection for adaptive change. In the dog, evidence has been found of repeat conservation across mammalian orders despite high mutation rates, suggesting strong stabilizing selection acting on these loci. In addition, it has been found that morphological differences between breeds of dog correlated with variations in repeat number [61]. Thus, in the presence of strong selection, significant repeat polymorphism can arise.

### Overall incidence of tandem repeat polymorphism

We noted that our estimate of polymorphism was higher when only clusters with a larger sample size were used (for example, 3.06% among 3,331 tandem repeats for which the UniGene cluster size was at least 200 sequences), indicating that our overall estimate is a lower estimate of the true frequency. Wren *et al*. [14] predicted that around 92% of polymorphic repeats in protein-coding regions would be a multiple of three nucleotides, which is concordant with the observation seen in Figure 1b. They experimentally confirmed 40% (17/42) of their predicted polymorphic protein-coding repeats within a sample of at least 60 chromosomes. Of the 249 unique repeat polymorphisms detected in our analysis, 56% were below the minimum threshold used by Wren *et al*. to predict polymorphism. Thus, while the method of Wren *et al*. is a useful prediction algorithm, it fails to predict many observed polymorphisms in shorter tandem arrays. Predicted polymorphism reflects the consequences of mutation, while actual polymorphism reflects the combination of mutation and subsequent selection pressures, and therefore the two approaches may well lead to different conclusions.

It is not surprising that a purely computational prediction will have false negatives, as it must protect against the problem of predicting too many false positives. We make the following assumptions: first, the Wren *et al*. prediction method only provides coverage of 44% (standard error 0.03) of tandem repeat polymorphisms, given that 56% of our variants were below their thresholds for polymorphism prediction; second, only 40% (standard error 0.08) of predicted repeats are actually polymorphic; third, there is one computationally predicted polymorphic tandem repeat per 23,000 nucleotides of protein-coding DNA [14]; and fourth, the average length of protein-coding DNA is 1,666 nucleotides (based on the UniGene dataset analyzed here). This then implies a revised estimate of estimated polymorphic tandem repeat copy-number variation to 1 in 25,000 nucleotides (with a 95% confidence interval of 17,911-43,066) [62], and that the average frequency of polymorphic tandem repeats in human proteins is 6%. The existence of annotation and experimental error may bias this upwards, while the existence of nonsense-mediated RNA decay may bias the estimate downwards.

Since 14.24% (42/295) of the polymorphisms were not a multiple of three nucleotides, up to 1% of proteins may contain frameshifting tandem repeat polymorphisms. It is likely that a much greater number of genes contain rarer frameshifting copy-number variants below the 1% frequency threshold used to define polymorphisms [63].

Our analysis confirms that tandem repeat variation is an important source of variation in many proteins. Much of this variation is of potential relevance to protein function and disease. A more thorough evaluation of the frequency of coding-sequence tandem repeat polymorphism will be possible once the resequencing of human exons from a panel of individuals becomes available. This will allow an unbiased assessment of the extent of common frameshifting tandem repeat variants. However, characterization of the frequency of rarer frameshifting tandem repeats will require larger sample sizes than typical current resequencing projects, as many repeats with large biological effects, such as frameshifts, are likely to occur at low frequencies. Thus, extensive resequencing or genotyping through large cohorts of individuals will be required in order to define their true incidence and to provide a clearer picture of the balance of mutational and selection pressures acting on the generation, fixation and elimination of tandem repeat copy-number variants in human genes.

## Materials and methods

### Detection of tandem repeats

Two files, Hs.seq.uniq and Hs.seq.all, from the UniGene database [15] build 172 were downloaded. Hs.seq.uniq was used as the template for tandem repeat detection and consisted of one sequence per UniGene cluster that contained the longest region of high-quality sequence data. Hs.seq.all consisted of a redundant set of gene-orientated sequences - that is, multiple sequences can correspond to the same gene cluster identifier. Tandem repeats detected in Hs.seq.uniq were defined as the queries. Tandem repeat blocks detected in Hs.seq.all using the queries were defined as the hits.

To ensure that there was no significant bias arising from expressed sequence tags (ESTs) of cancerous origin, we eliminated these sequences from our results by using the TissueInfo [64] classification of EST libraries (December 2002).

Tandem repeats are often complex patterns and it was found that repeats were often detected as smaller sub-patterns when using a lower minimum score to report a repeat. This occurred for the 69-nucleotide repeat in *MUC2 *for instance, where the repeat unit was detected as a series of six- and three-nucleotide repeat units. As we wanted to detect the largest range of repeats possible while retaining repeat patterns that were correct, we decided to retain all repeats detected under default parameter settings and then to search for repeats using more sensitive parameters. Only repeats detected in the latter search that did not overlap with those in the former were included.

Tandem repeats were first detected in Hs.seq.uniq using the Tandem Repeats Finder (TRF) program version 3.21 [65] with default parameters for repeat detection. A minscore of 12 instead of 50 was used the second time round, which corresponds to a minimum of three copies of a 2-nucleotide repeat as an example. The TRF detection cutoff of 12 was deliberately chosen to be low: this was motivated by the desire to determine the level of repeat variation in all repeats, regardless of their mutational origin. Thus, of the repeats we investigated, 98% (87,787/89,243) had scores below the TRF default score of 50. Of the variants detected, 67% (167/249) had a TRF score below 50. Thus, searches for variant tandem repeats need to consider low copy-number repeats, as well as those high copy-number repeats which are more likely to be variant. For shorter arrays to be reported by TRF, they will need to be 100% homogeneous to be detectable. Clearly, there may be other insertions or deletions among short inexact repeat arrays that we have not detected. Sequences lacking 25 nucleotides of flanking sequence on both sides of the detected tandem repeat block were omitted from further analysis.

We restricted our analysis to variability among protein-coding repeat sequences. Definitions of coding sequence (CDS) start and stop points were taken from the sequence header of the Hs.seq.uniq sequences in UniGene. Sequences lacking CDS information and tandem repeat sequences that did not lie exclusively within coding regions were not included. Mononucleotide tandem repeats were excluded from the analysis, as we considered the probability of detecting sequence errors too great [66].

### Detection of tandem repeat variation

Similarity of the tandem repeat region within the Hs.seq.uniq representative to the same region within other sequences within the cluster was assessed by matching up the corresponding sequences using their 25-nucleotide flanks. Length differences were detected by comparing the length of the representative tandem repeat block to that of the other sequences in the cluster.

Detected repeat blocks thus have the following properties: a 25-nucleotide flanking sequence on both sides (which is used to align repeat blocks from different sequences in the cluster), and they belong to a cluster containing more than one sequence overlapping the tandem repeat sequence block and its 25-nucleotide flanks.

Detected variants were screened to ensure that they represented length variation arising as copy-number differences in genomic DNA rather than intron retention or alternative splicing: Only length variations that corresponded to a length difference that was a multiple of the repeat unit were selected. For this set, tandem repeats were detected in the variant sequence and checked to ensure that the observed copy-number agreed with the expected one, given the length of the hit block and the length of the repeat unit.

We calculated the gene diversity (or heterozygosity) as



where *Pi *is the frequency of the *i*th of *k *repeat lengths at a locus ([67] and see Additional data file 2).

### Gene Ontology (GO) data

To test the hypothesis that the number of genes with tandem repeat variation is elevated in genes involved in defense-related processes, the term 'defense response' (GO:0006952) was selected from GO. Human UniGene clusters linked to GO terms and their hierarchies were obtained by linking LocusLink to both UniGene and GO and also by linking UniGene to EMBL and then linking, via the EMBL accessions, to UniProt and thence to GO. Links were subsequently completed by adding links to all parent GO terms for each GO term using the GO_GRAPH_PATH and GO_TERM tables from the Gene Ontology database (dated 1 July 2004). By cross-referencing our GO term of interest with the file linking GO to UniGene, we were able to assign a binary classification (yes/no related to our GO term of interest) to each UniGene cluster. This allowed us to statistically assess the differences in the levels of variation between genes related and not related to the defense response. Significant terms were corrected for multiple testing using the Bonferroni method. Statistical analysis was carried out in STATA 8.

## Additional data files

The following additional data is available with the online version of this paper. Additional data file 1 is a table listing the 295 repeat variants (spanning 218 UniGene clusters) detected in our analysis, with information on the repeats and a description of the cluster representative sequence. Additional data file 2 contains block lengths of repeats grouped into 249 unique repeat loci. For each locus, the heterozygosity of the repeat length allele frequencies has been calculated. Additional data file 3 contains data used for Figure 3. Counts of variant and invariant repeats of different unit lengths and copy-numbers are tabulated.

## Supplementary Material

Additional data file 1 295 repeat variants (spanning 218 UniGene clusters) detected in our analysis, with information on the repeats and a description of the cluster representative sequence. Click here for file

Additional data file 2 Block lengths of repeats grouped into 249 unique repeat loci. For each locus, the heterozygosity of the repeat length allele frequencies has been calculated. Click here for file

Additional data file 3 Data used for Figure 3. Counts of variant and invariant repeats of different unit lengths and copy-numbers are tabulated. Click here for file

## Figures and Tables

**Table 1:** GO analysis of repeat variants

Term	GO id	Variants		Non-variants		Statistical tests			
							
		Linked	Not linked	Linked	Not linked				
**(a) **Primary hypothesis									
Defense response	GO:0006952	9	150	475	7,979	Chi-squared test 0.98		Logistic regression 0.88	
**(b) **All level 4									
						Fishers exact		Logistic regression	
						Fishers exact	Bonferroni	Logistic regression	Bonferroni
**Most significant terms**									
Protein binding	GO:0005515	45	114	1,354	7,100	<0.00001	<0.0006	<0.00001	<0.0006
Morphogenesis	GO:0009653	28	131	783	7,671	0.001	0.064	0.001	0.064
Intracellular	GO:0005622	93	66	3,845	4,609	0.001	0.064	0.004	0.256
Transcription cofactor activity	GO:0003712	10	149	166	8,288	0.002	0.128	<0.00001	<0.0006
RNA polymerase II transcription factor activity	GO:0003702	9	150	150	8,304	0.003	0.192	0.001	0.064
Protein serine/threonine phosphatase complex	GO:0008287	3	156	19	8,435	0.007	0.448	<0.00001	<0.0006
Helicase activity	GO:0004386	6	153	93	8,361	0.01	0.64	0.007	0.448
Structural constituent of epidermis	GO:0030280	2	157	7	8,447	0.011	0.704	0.001	0.064
Regulation of physiological process	GO:0050791	37	122	1,339	7,115	0.016	1.024	0.014	0.896
Death	GO:0016265	11	148	272	8,182	0.02	1.28	0.01	0.64
Pattern specification	GO:0007389	2	157	17	8,437	0.047	3.008	0.013	0.832
Antigen binding	GO:0003823	2	157	18	8,436	0.052	3.328	0.021	1.344
